# Immune cells harbor their own microbiome-derived metabolome: a new layer of immunometabolic regulation

**DOI:** 10.3389/fimmu.2026.1852583

**Published:** 2026-07-28

**Authors:** Lisa Daley-Bauer, Luke Flantzer, Shuya Kyu, Anyce Godoy, Jaclyn Weinberg, Vincent C. Marconi, Dean P. Jones, Souheil-Antoine Younes

**Affiliations:** 1 Pathology Advanced Translational Research Unit (PATRU), Department of Pathology and Laboratory Medicine, Emory University School of Medicine, Atlanta, GA, United States; 2 Division of Infectious Diseases Emory University School of Medicine and Department of Global Health, Emory University Rollins School of Public Health, Atlanta, GA, United States; 3 Division of Pulmonary, Allergy, Critical Care and Sleep Medicine, Department of Medicine, Emory University School of Medicine, Atlanta, GA, United States

**Keywords:** aryl hydrocarbon receptor, gut-derived bacterial metabolites, intracellular microbiome metabolome, P-cresol sulfate, SLCO4A1/OATP4A1

## Abstract

Current models of microbiome, immune crosstalk center on extracellular receptor-mediated signaling, yet a critical observation challenges this paradigm: intracellular concentrations of gut-derived bacterial metabolites (GDBMs) in CD4^+^ T cells do not correlate with paired plasma levels, and it is intracellular, not circulating, GDBM burden that associates with metabolic pathway disruption and immune senescence. Here we propose the concept of an intracellular microbiome metabolome: a pool of aromatic GDBMs actively accumulated through carrier-mediated transport, retained through transcriptional suppression of efflux transporters, and integrated into host metabolic networks where metabolites directly engage intracellular senescence pathways. Using p-cresol sulfate (PCS) as a mechanistic prototype, we provide transcriptomic, proteomic, and metabolomic evidence implicating SLCO4A1/OATP4A1 as the primary entry transporter, whose suppression following PCS exposure creates a feed-forward intracellular retention loop. Once accumulated, PCS functions as a direct agonist of the aryl hydrocarbon receptor (AhR), engaging five downstream effector programs, TGF-β/SMAD signaling, Wnt/β-catenin reprogramming, Foxp3-dependent Treg induction, Notch dysregulation, and PTGS2/COX-2 induction with coordinate HPGD suppression driving PGE_2_ excess via EP2/EP4/cAMP/CREM, that converge on mTOR suppression, glycolytic collapse, and mitochondrial dysfunction. This metabolic collapse in turn activates the integrated stress response (ISR) as a downstream consequence, driving p16/CDKN2A and p21/CDKN1A induction and the full immunometabolic signature of accelerated CD4^+^ T cell aging. The plasma intracellular dissociation explains why circulating GDBM levels have failed to predict immune outcomes in HIV-1 infection, chronic kidney disease, and aging, and positions intracellular GDBM quantification as the biologically relevant exposure metric. We discuss three therapeutic intervention layers: reduction of microbial metabolite production, blockade of SLCO4A1-mediated entry and efflux suppression, and targeting the AhR signaling axis with downstream metabolic and ISR consequences.

## 1 Introduction

The gut microbiome generates a chemically diverse repertoire of metabolites that enter the systemic circulation and profoundly shape the host immune system (1, 2). As examples, short-chain fatty acids (SCFAs) regulate peripheral Treg homeostasis and macrophage activation (3–5), secondary bile acids modulate innate immune responses through FXR and TGR5 (6, 7), and tryptophan-derived indole metabolites activate the aryl hydrocarbon receptor (AhR) to shape T cell fate (8, 9). Gut dysbiosis, a feature of chronic kidney disease (CKD), HIV-1 infection, inflammatory bowel disease, and aging, disrupts this metabolite landscape, elevating circulating levels of aromatic uremic metabolites including p-cresol sulfate (PCS), indoxyl sulfate (IS), and phenylacetylglycine (PAG), which is consistently associated with impaired CD4^+^ T cell function, premature immune senescence, and accelerated immune aging (10, 11). Despite this clinical relevance, the mechanisms by which these metabolites drive immune dysfunction remain incompletely understood.

**Figure 1:**
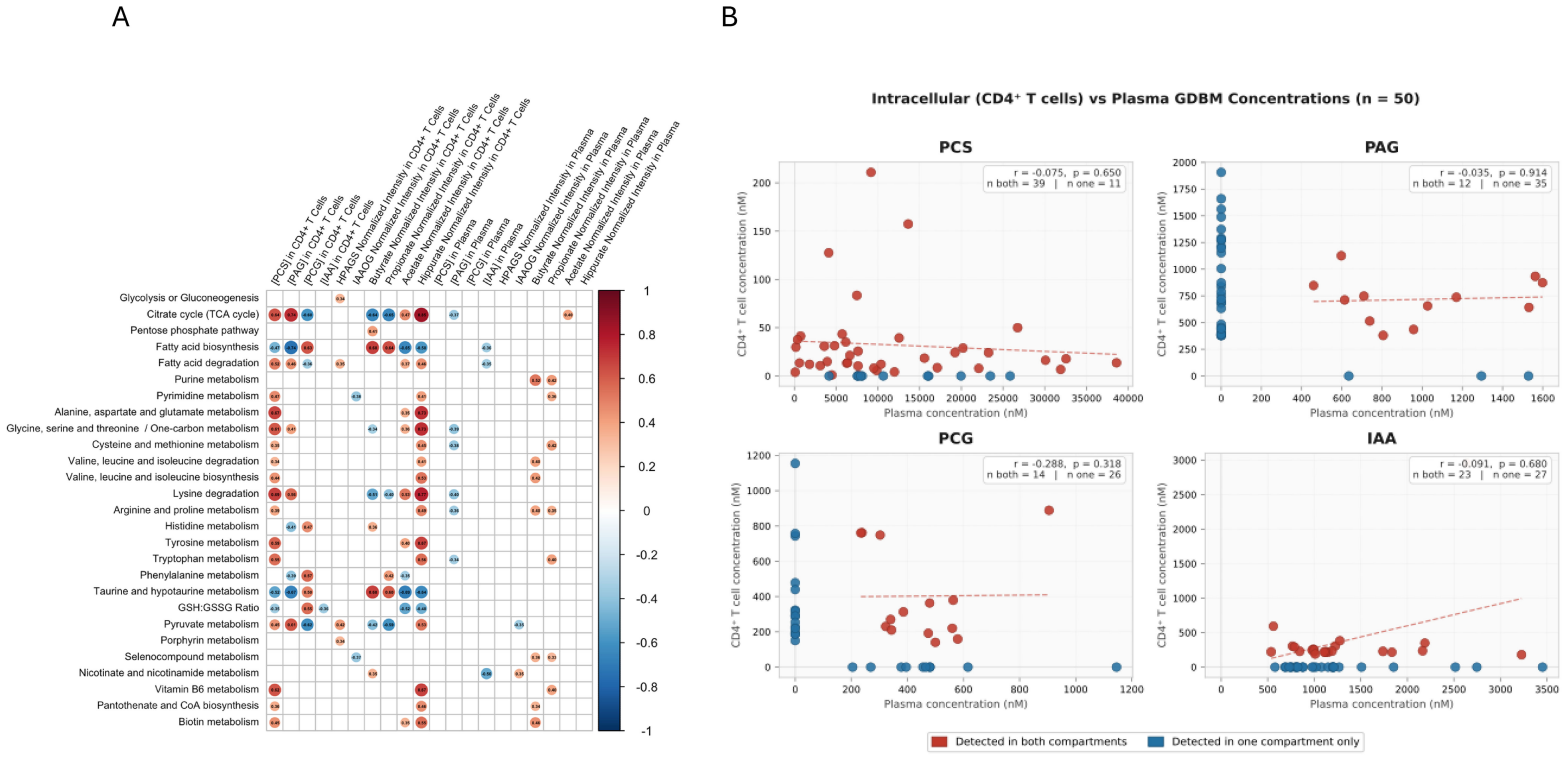
Intracellular GDBMs associate with metabolic pathway remodeling in CD4^+^ T cells from ART-suppressed PLWH. **(A)** Untargeted and targeted metabolomic profiling of purified CD4^+^ T cells from 26 aviremic HIV-1–infected immune responders reveal correlations between intracellular microbiome-derived metabolites (GDBMs) and major metabolic pathways. Metabolites with quantified concentrations (in brackets) and those measured by relative intensity show distinct patterns of positive (red) and negative (blue) associations. Positive correlations indicate accumulation and suggest dysregulation of metabolic pathways linked to immune dysfunction. **(B)** Comparison of plasma and intracellular concentrations of GDBMs in matched samples demonstrates a lack of direct correlation between circulating and CD4^+^ T cell–associated metabolite levels. Distinct compartmentalization is observed across metabolites: PAG and PCG are detected more frequently intracellularly than in plasma, suggesting selective uptake or intracellular retention, whereas IAA is broadly detected in plasma but less frequently in CD4^+^ T cells, indicating limited cellular access. PCS shows widespread detection in plasma with partial intracellular enrichment. Together, these findings highlight that intracellular GDBM abundance is not determined by systemic exposure alone but reflects cell-intrinsic transport and retention mechanisms.

Current models of microbiome–immune communication center on extracellular receptor-mediated signaling, in which circulating microbial metabolites engage cell-surface or nuclear receptors to trigger downstream cascades. This receptor-centric paradigm has generated fundamental mechanistic insights but captures only part of the overarching framework. A critical observation challenges the completeness of this narrative: intracellular concentrations of GDBMs in CD4^+^ T cells do not correlate with paired plasma levels and cannot be predicted from systemic metabolite abundance (12). If extracellular receptor engagement were the primary mechanism, plasma concentrations should be the relevant exposure variable, yet it is intracellular GDBM burden, not circulating levels, that correlates significantly with metabolic pathway disruption and immune senescence markers in primary human CD4^+^ T cells (12) (**Figure 1B**). Moreover, several key effectors implicated in GDBM biology, the Aryl Hydrocarbon Receptor (AhR), which require intracellular ligand binding for nuclear translocation (13) and GCN2 (14) and sense cytoplasmic uncharged tRNAs, are fundamentally intracellular sensors that cannot be activated from the cell exterior. These observations converge on a conclusion the receptor-centric model cannot accommodate: the primary site of GDBM action in immune cells is intracellular.

Yet the field has not articulated a mechanistic framework for intracellular GDBM biology in immune cells. Which transporters mediate GDBM entry into T cells and other immune subsets, as distinct from the well-characterized renal OAT1/OAT3 machinery (15, 16), is largely unknown. How intracellular GDBMs engage specific stress-sensing pathways, and whether these mechanisms extend beyond PCS to structurally related aromatic metabolites, has not been defined. Most critically, the mechanistic connection between intracellular GDBM accumulation and canonical immunosenescence programs, including p16/CDKN2A and p21/CDKN1A induction, AhR-driven transcriptional reprogramming, and mitochondrial dysfunction, has not been established.

**Figure 2:**
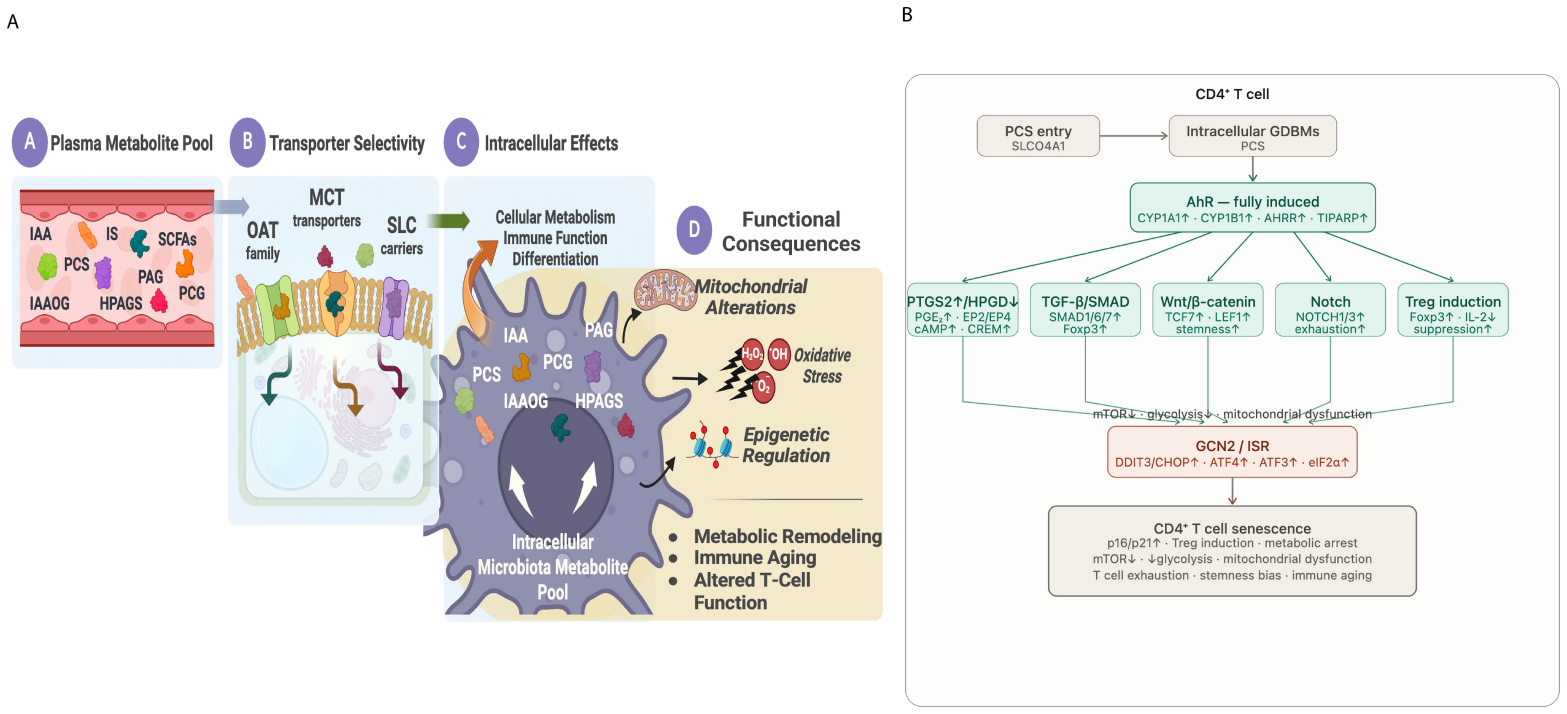
Intracellular retention of microbiome-derived metabolites drives CD4^+^ T cell immune aging. **(A)** Gut microbiota-derived metabolites (GDBMs) circulate in plasma and enter CD4^+^ T cells through selective transporters, generating a distinct intracellular metabolite pool that drives metabolic, epigenetic, and functional reprogramming. **(B)** PCS preferentially enters via SLCO4A1 and accumulates intracellularly, inducing coordinated activation of AhR, PGE_2_, and integrated stress response (ISR) pathways, leading to CD4^+^ T cell senescence, immune dysfunction, and metabolic remodeling.

Here we propose the concept of an intracellular microbiome metabolome within immune cells: a pool of gut-derived bacterial metabolites that are actively accumulated through carrier-mediated transport, retained through transcriptional suppression of efflux transporters, and integrated into host metabolic networks where they directly engage intracellular stress-response and senescence pathways (**Figure 2A**). As illustrated, this concept can be understood as a four-stage process operating between the systemic circulation and immune cell function. First, a chemically diverse plasma metabolite pool (Panel A), comprising aromatic conjugates including PCS, PAG, PCG, IAA, IAAOG, and HPAGS alongside SCFAs, circulates in the bloodstream following gut microbial production and hepatic biotransformation. Second, cellular entry is not passive or uniform but is governed by transporter selectivity (Panel B): aromatic conjugated GDBMs are imported via organic anion transporter family members including SLCO4A1/OATP4A1 and related SLC carriers, while SCFAs access the cell through a mechanistically distinct route via monocarboxylate transporters of the MCT family. This selectivity, determined by the chemical class, conjugation state, and transporter expression profile of the immune cell, implies that the intracellular GDBM composition is actively shaped rather than passively mirroring the plasma pool. Third, once inside the immune cell, these metabolites are not inert bystanders. They establish an intracellular microbiota metabolite pool (Panel C) that directly reprograms cellular metabolism, immune function, and differentiation, engaging pathways that cannot be accessed from the extracellular compartment. Fourth and finally, this intracellular accumulation drives a coordinated set of functional consequences (Panel D) that together define the immunometabolic collapse at the center of this framework: mitochondrial alterations reflecting bioenergetic failure, oxidative stress from disrupted redox homeostasis, and epigenetic reprogramming that reshapes transcriptional programs governing immune cell identity and longevity, collectively producing metabolic remodeling, immune aging, and altered T cell function. The critical insight illustrated in the figure is that the critical biological event is not what circulates but what accumulates inside the immune cell, and that the transporter selectivity of Panel B is as determinative of immune fate as the composition of the plasma pool in Panel A. Using p-cresol sulfate (PCS) as a mechanistic prototype, we present transcriptomic and proteomic evidence implicating SLCO4A1/OATP4A1 (solute carrier organic anion transporter family member 4A1/organic anion transporting polypeptide 4A1) as a primary entry transporter for PCS in CD4^+^ T cells, an inference supported by its significant downregulation following PCS exposure in both bulk RNA sequencing (RNA-seq), single-cell RNA sequencing (scRNA-seq), and proteomics (12), consistent with a self-reinforcing intracellular retention mechanism. Once accumulated intracellularly, PCS functions as a direct agonist of the intracellular aryl hydrocarbon receptor (AhR), inducing the full spectrum of AhR transcriptional targets including cytochrome P450 family 1 subfamily A member 1 (CYP1A1) and cytochrome P450 family 1 subfamily B member 1 (CYP1B1), and engaging five downstream effector programs: transforming growth factor-β/SMAD (small mothers against decapentaplegic) signaling, Wnt/β-catenin/TCF7 (T cell factor 7) reprogramming, forkhead box P3 (Foxp3)-dependent regulatory T cell (Treg) induction, Notch pathway dysregulation, and upregulation of prostaglandin-endoperoxide synthase 2 (PTGS2)/cyclooxygenase-2 (COX-2) with coordinate suppression of hydroxyprostaglandin dehydrogenase 15-(NAD^+^) (HPGD), the primary enzyme responsible for prostaglandin E_2_ (PGE_2_) degradation, driving intracellular and extracellular PGE_2_ accumulation via a feed-forward loop through E-prostanoid receptor 2 and 4 (EP2/EP4)/cyclic adenosine monophosphate (cAMP)/cAMP-responsive element modulator (CREM) signaling. These five AhR-driven programs converge on mechanistic target of rapamycin (mTOR) suppression, glycolytic collapse, and mitochondrial dysfunction, which in turn activates the general control non-derepressible 2/integrated stress response (GCN2/ISR) as a downstream metabolic consequence, driving cyclin-dependent kinase inhibitor 2A (p16/CDKN2A) and cyclin-dependent kinase inhibitor 1A (p21/CDKN1A) induction, Treg enrichment, and CD4^+^ T cell immune aging (**Figure 2B**).

Intracellular GDBM accumulation — not plasma levels — is the critical exposure, explaining why systemic metabolite concentrations have failed to consistently predict immune outcomes across clinical cohorts. This framework builds on and extends three prior conceptual contributions. The SCFA literature established that GDBMs can enter immune cells via MCT transporters and act as intracellular epigenetic regulators through HDAC inhibition (5) providing key precedent for intracellular GDBM biology but limited to a single metabolite class and mechanism. The IDO/GCN2/Treg axis demonstrated that amino acid depletion activates GCN2 in T cells with immunosuppressive consequences (17, 18) but through extracellular tryptophan catabolism by neighboring cells rather than cell-autonomous intracellular metabolite accumulation. The uremic toxin field has documented immune dysfunction driven by PCS and IS in CKD (15, 19, 20) but has remained plasma-centric without defining the intracellular sensing mechanisms. Our framework unifies these threads by demonstrating that aromatic GDBMs, as sulfated and conjugated organic anions, gain cellular entry via OATP-family transporters including SLCO4A1/OATP4A1 (21, 22) accumulate intracellularly due to coordinate efflux suppression, and activate GCN2/ISR as a downstream consequence of intracellular metabolic stress rather than through competitive leucine displacement at the point of entry. We further show that this mechanism extends beyond PCS: multiple structurally distinct GDBMs including PAG, PCG, IAA, and SCFAs accumulate within CD4^+^ T cells and perturb overlapping metabolic networks (12) (**Figure 1A**) establishing generalizability as a core feature of the intracellular microbiome metabolome concept. In addition, across all metabolites examined that we quantitate by targeted metabolic analysis (PCS, PCG, PAG, and IAA), we observed no significant correlation between plasma and intracellular concentrations, despite consistent detectability in one or both compartments (**Figure 1B**). For PAG, only 15 plasma samples were above detection, whereas PAG was detected in CD4^+^ T cells in 44 cases, strongly suggesting that PAG is not merely passively inherited from plasma levels but can accumulate or persist intracellularly. A similar pattern is observed PCG, which was detected less frequently in plasma than in CD4^+^ T cells, again supporting the idea that intracellular immune cells may harbor a distinct metabolite pool enriched for selected GDBMs. Importantly, targeted quantification of PCS, PCG, PAG, and IAA was performed by method of standard addition, in which calibration curves are constructed within each individual sample matrix, directly correcting for matrix-specific ion suppression, protein binding interference, and extraction efficiency differences between plasma and cell lysates. Metabolite identity was further confirmed by MS2 fragmentation, and internal standards were included in all extractions. The differential detection frequencies observed between plasma and intracellular compartments therefore reflect genuine compartment-specific metabolite abundance rather than matrix-dependent analytical artifacts. In contrast, IAA was detected in all plasma samples but in only 23 CD4^+^ T cell samples, indicating that abundant systemic exposure does not necessarily translate into intracellular access. PCS shows an intermediate pattern: it was detected in all plasma samples and in 39 CD4^+^ T cell samples, consistent with broad systemic availability but selective intracellular accumulation. Together, these findings support the concept that immune cells maintain their own intracellular microbiome-derived metabolome, and that the biological impact of a given metabolite depends not simply on its presence in plasma, but on its ability to gain intracellular access and persist within the target cell. Collectively, these data highlight a critical disconnect between circulating and intracellular metabolite levels and underscore the importance of studying cell-associated metabolite dynamics to understand immune regulation.

## 2 Sources, biotransformation, and chemical diversity of gut-derived bacterial metabolites

The gut microbiome generates its metabolite repertoire primarily through two catabolic routes: fermentation of dietary complex carbohydrates and degradation of dietary amino acids. Fermentation of non-digestible fibers by saccharolytic commensals, principally *Firmicutes* and *Bacteroidetes*, produces SCFAs, including acetate, propionate, and butyrate, at millimolar concentrations in the colon (1, 23, 24). SCFAs are the best-characterized immunomodulatory GDBMs and establish the foundational principle that microbial metabolites can enter host cells, via monocarboxylate transporters MCT1 and MCT4, and directly reprogram immune cell gene expression through intracellular mechanisms including histone deacetylase inhibition (4, 5). In parallel, proteolytic bacteria metabolize dietary aromatic amino acids along distinct pathways that generate a structurally diverse class of metabolites with markedly different chemical properties and as discussed, different fates within immune cells. Bacterial deamination and decarboxylation of tyrosine and phenylalanine produces p-cresol and phenylacetic acid respectively (25, 26), while tryptophan is catabolized by microbial tryptophanases to indole and downstream indole derivatives including indole-3-acetic acid (IAA) and indole-3-propionic acid (27, 28). The relative output of saccharolytic versus proteolytic fermentation is therefore directly determined by dietary macronutrient composition, explaining why high-protein, low-fiber diets consistently elevate circulating aromatic GDBM levels (29).

**Figure 3:**
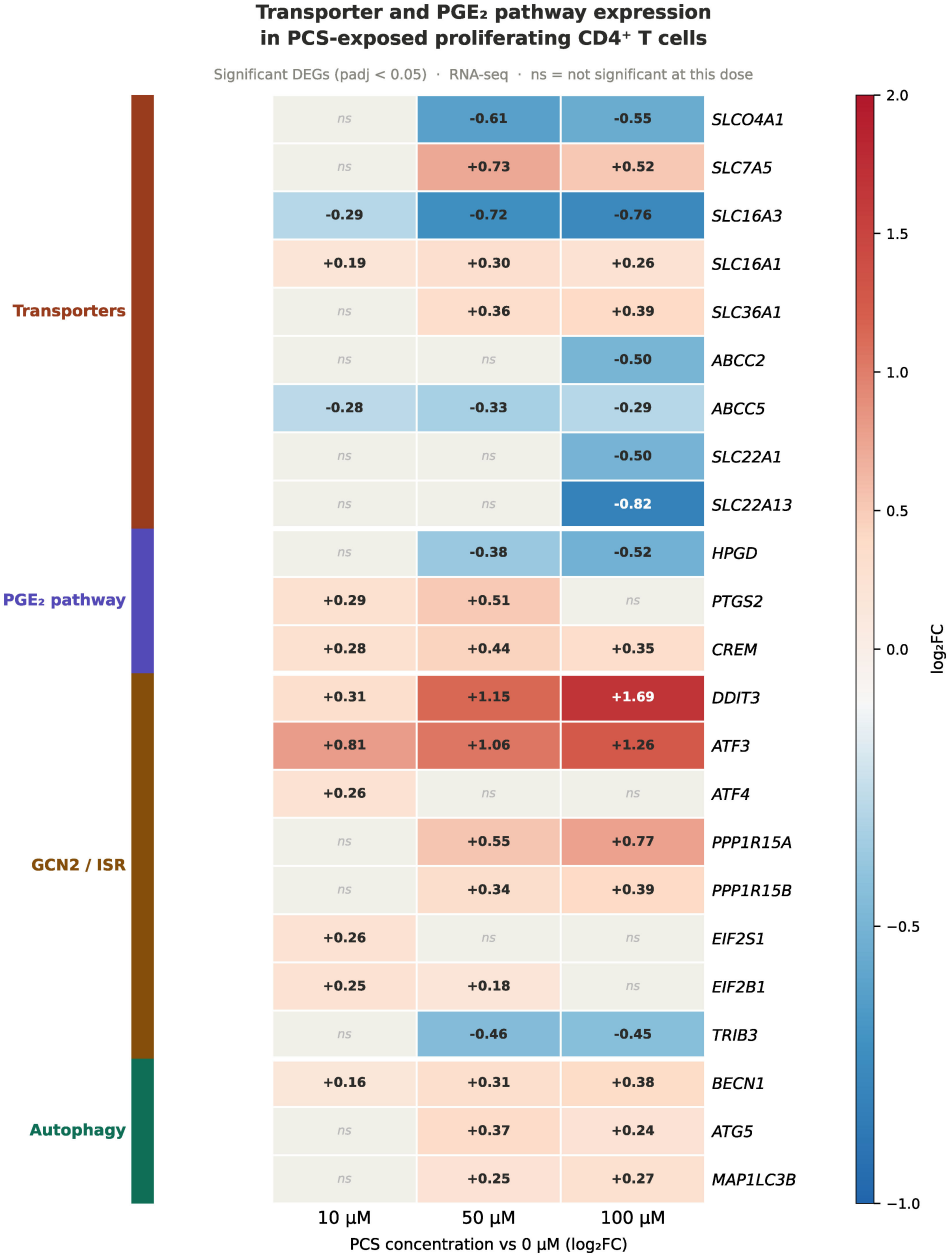
PCS induces a dose-dependent transcriptional program in proliferating CD4^+^ T cells across transporter, PGE_2_, GCN2/ISR, and autophagy pathways. Bulk RNA-seq analysis of proliferating (CTV^−^) CD4^+^ T cells from 5 healthy donors stimulated with anti-CD3/CD28 for 6 days in the presence of increasing PCS concentrations (0, 10, 50, 100 μM) reveals coordinated modulation of transporter pathways, PGE_2_ pathway, integrated stress response (ISR), and autophagy pathways. Differential expression is shown as log_2_ fold change relative to untreated controls, highlighting dose-dependent induction of stress and metabolic programs and suppression of transporter and clearance mechanisms. Baseline normalized expression levels for all transporters shown, confirming detectable expression in activated CD4^+^ T cells prior to PCS exposure, are provided in the companion data paper Da Silva et al. (2026) (12).

Before reaching the systemic circulation, microbial aromatic metabolites absorbed from the intestine undergo extensive phase II biotransformation in the liver, a step that is critical for understanding their subsequent cellular biology. Hepatic SULT1A1 is the primary sulfotransferase responsible for conjugating p-cresol to generate PCS, while UDP-glucuronosyltransferases generate PCG as a minor competing product whose relative abundance shifts with declining renal function in advanced CKD (30, 31). Phenylacetic acid, derived from phenylalanine by gut bacteria, is conjugated in hepatic and renal mitochondria by glycine N-acyltransferase to generate phenylacetylglycine (PAGgly), alongside glutamine conjugation to PAGln (32, 33). Indole is hydroxylated in the liver by CYP2E1 to yield indoxyl, which is then sulfated by SULT1A1 to generate indoxyl sulfate (IS) (34), while IAA circulates largely unconjugated or as IAA O-glucuronide (IAAOG) (27). These conjugation reactions were classically interpreted as detoxification steps facilitating renal clearance, and indeed, conjugated GDBMs are normally excreted renally, explaining their dramatic accumulation in chronic kidney disease where clearance is impaired (10). However, the conjugated forms are not biologically inert. Sulfation and glucuronidation alter the charge, hydrophilicity, and protein-binding properties of these metabolites in ways that profoundly influence their tissue distribution and cellular uptake. Approximately 95% of circulating PCS and IS is bound to albumin at Sudlow site II, leaving only a small free fraction available for passive membrane diffusion (31, 35), a property that effectively mandates carrier-mediated transport for cellular entry. Furthermore, conjugated forms such as PCS are poor substrates for the efflux transporters (ABCC2/MRP2, ABCC5/MRP5) that would otherwise clear them from cells, transporters whose expression in CD4^+^ T cells is low at baseline and is further suppressed by PCS exposure in our dataset (**Figure 3**), creating conditions for intracellular retention that non-conjugated precursors would not share. The resulting circulating GDBM pool is chemically heterogeneous but shares important structural features relevant to immune cell uptake. Aromatic GDBMs, PCS, IS, PAG, PCG, IAA, IAAOG, are all derived from aromatic amino acid precursors (tyrosine, phenylalanine, tryptophan) and, following hepatic phase II conjugation, circulate as sulfated or glucuronidated organic anions. These conjugated forms are recognized substrates of OATP-family transporters, including SLCO4A1/OATP4A1, which mediates sodium-independent uptake of estrone sulfate, estradiol-17β-glucuronide, prostaglandins, and structurally related amphipathic organic anions (16, 21, 22). This shared chemical scaffold, amphipathic, negatively charged at physiological pH, and largely protein-bound in plasma, is precisely the substrate class recognized by OATP-family transporters including SLCO4A1/OATP4A1. By contrast, SCFAs, the other major immunologically active GDBM class, are monocarboxylates entering cells via MCT1/MCT4 through an entirely distinct mechanism (36, 37).

To determine whether intracellular GDBM accumulation is biologically meaningful and functionally distinct from systemic exposure, we performed correlation analyses between intracellular GDBM concentrations in CD4^+^ T cells and major cellular metabolic pathway signatures inferred from targeted metabolomic profiling and compared these with correlations obtained using paired plasma concentrations of the same metabolites in the same individuals (**Figure 1**). A striking and asymmetric pattern emerged. When GDBM concentrations were measured inside CD4^+^ T cells, robust and predominantly positive correlations were observed across a broad spectrum of central metabolic pathways, including glycolysis and gluconeogenesis, the TCA cycle, fatty acid biosynthesis and degradation, multiple amino acid metabolism pathways, pyruvate metabolism, and redox balance as indexed by the GSH: GSSG ratio. This pattern of positive correlations across interconnected bioenergetic pathways is most consistent with accumulation of metabolic intermediates reflecting impaired flux and metabolic bottlenecks rather than pathway activation, a signature compatible with mitochondrial stress, altered oxidative metabolism, and bioenergetic failure. Intracellular PCS showed the broadest and strongest correlation profile, associating with the greatest number of pathways, consistent with its role as the mechanistic prototype of intracellular GDBM biology. Other intracellular GDBMs, PAG, PCG, IAA, IAAOG, HPAGS, and SCFAs, showed overlapping but partially distinct correlation signatures, establishing that multiple structurally diverse GDBMs converge on shared metabolic disruption networks within the same cell. Notably, PCG showed a partially opposing correlation pattern relative to PCS, consistent with the possibility that glucuronide versus sulfate conjugation alters intracellular routing or functional impact. By stark contrast, when the same analyses were repeated using plasma concentrations of the identical metabolites in the same individuals, these metabolic pathway correlations were almost entirely absent. The right portion of the correlation matrix is largely empty, with only scattered weak associations. This dissociation, robust intracellular associations in the complete absence of plasma associations, establishes that circulating GDBM levels do not reflect the intracellular metabolic state of CD4^+^ T cells, and provides the strongest evidence that the intracellular microbiome metabolome is a biologically distinct entity rather than a passive reflection of systemic metabolite abundance.

Gut dysbiosis, a feature common to CKD (10, 19, 20), HIV-1 infection (38–41), aging (10, 42, 43), and inflammatory bowel disease (9, 44, 45), selectively amplifies the aromatic GDBM pool. Dysbiotic microbiomes are characterized by depletion of saccharolytic fiber-fermenting commensals and expansion of proteolytic taxa with high tyrosine lyase and tryptophanase activity (25–28), shifting microbial metabolism toward aromatic amino acid catabolism (10, 19, 29, 46). In PLWH, gut dysbiosis is driven by HIV-associated damage to the intestinal epithelium (47), microbial translocation (48), and persistent immune activation even during effective ART (49–51), resulting in elevated plasma PCS and enrichment of PCS within CD4^+^ T cells relative to immunologically healthy individuals (11). In CKD, impaired renal clearance compounds dysbiosis-driven overproduction, producing total plasma concentrations of PCS and IS that reach 100–150 μM in maintenance hemodialysis patients and can substantially exceed this range in individual patients with end-stage renal disease (52, 53). Crucially, the finding that intracellular PCS concentrations in CD4^+^ T cells do not correlate with plasma levels (11, 12) argues that dysbiosis-driven changes in circulating GDBMs are necessary but not sufficient to predict intracellular immune cell burden: the transport and retention mechanisms described in Section 3 are equally determinative.

## 3 Transport mechanisms governing intracellular GDBM accumulation in immune cells

For gut-derived bacterial metabolites to directly influence intracellular immune cell biology, they must first cross the plasma membrane, a requirement that is non-trivial for the aromatic conjugated GDBMs that are the focus of this review. PCS, IS, PAG, and related metabolites are polar, negatively charged at physiological pH, and largely protein-bound in plasma, properties that collectively preclude significant passive membrane diffusion (10). Consistent with regulated rather than passive uptake, intracellular PCS concentrations in CD4^+^ T cells from PLWH range from undetectable to over 200 nM across individuals yet show no correlation with paired plasma levels, and are enhanced by TCR activation indicating that cellular metabolic state, not extracellular concentration gradient, governs accumulation (12). Although the extracellular PCS concentrations used *in vitro* (10–100 μM) exceed measured plasma free fractions, calibration experiments demonstrate that exposure to 50–100 μM PCS results in intracellular cell-associated concentrations of approximately 4–16 nM, within the range detected ex vivo in CD4^+^ T cells of virally suppressed PLWH (undetectable to ~210 nM; mean ~4–101 nM across PCS quartile groups). TCR stimulation further enhances PCS cell association, consistent with activation-dependent regulated uptake. These data support the physiological relevance of the *in vitro* transcriptional program described. These observations establish that immune cells actively regulate their intracellular GDBM content through carrier-mediated mechanisms. Three transporter families are implicated: organic anion transporters of the SLCO/OATP family, with SLCO4A1/OATP4A1 as the primary candidate for aromatic conjugate uptake such as PCS, the SLC22 OAT family, and monocarboxylate transporters (MCTs) of the SLC16 family, with their relative contributions varying by metabolite class and immune cell type. In renal tubular epithelium, the SLC22 organic anion transporter family, particularly OAT1 (SLC22A6) and OAT3 (SLC22A8), constitutes the primary uptake machinery for PCS and IS at the basolateral membrane, mediating their tubular secretion and urinary clearance (15, 16, 54). Whether OAT family members are expressed and functionally active in lymphocytes and other immune cells is less well established. Expression profiling across 50 xenobiotic transporter genes in human tissues identified restricted OAT expression outside classic barrier organs (55) and the recent finding that SLC22A11 (OAT4) inserts IS and PCS directly into the plasma membrane of non-renal cells (56) raises the possibility of OAT-independent membrane association. However, OAT-mediated transport into the cytoplasm, as distinct from membrane insertion, has not been demonstrated in primary T cells, and the kinetics of PCS accumulation we observe are not consistent with constitutive OAT activity (57). OAT-mediated uptake may therefore contribute to GDBM accumulation in certain immune cell types or disease contexts but is unlikely to be the primary route in activated T cells. Our transcriptomic analysis of PCS-exposed CD4^+^ T cells identifies SLCO4A1/OATP4A1 as the most compelling entry transporter candidate for PCS in immune cells. SLCO4A1 encodes OATP4A1, a member of the solute carrier organic anion transporter (SLCO/OATP) superfamily that mediates sodium-independent, carrier-mediated uptake of amphipathic organic anions including estrone-3-sulfate, estradiol-17β-glucuronide, prostaglandins, and related sulfated and glucuronidated metabolites, a substrate class chemically congruent with PCS and other aromatic GDBMs (21, 22, 58). SLCO4A1 expression has been detected in primary human immune cells, including monocytes, macrophages, and antigen-presenting cells (59, 60) establishing precedent for OATP-mediated transport in cells of hematopoietic lineage. Critically, SLCO4A1 is significantly downregulated by PCS exposure at both the transcriptional and protein levels, with confocal microscopy in primary CD4^+^ T cells confirming significant reduction of SLCO4A1 protein following PCS exposure (Da Silva et al., 2026) (12) (**Figure 3**). This transcriptional suppression following PCS exposure is the mechanistic hallmark of a feed-forward retention loop: SLCO4A1 mediates initial PCS entry, and its subsequent downregulation by intracellular PCS impairs efflux of the same substrate class, promoting progressive intracellular accumulation. This logic, transporter-mediated entry followed by transcriptional suppression of that same transporter, parallels the well-characterized downregulation of renal OAT transporters by their uremic toxin substrates in CKD, and is consistent with a cell-autonomous trapping mechanism operating independently of extracellular PCS concentrations. Direct transport assays in primary CD4^+^ T cells will be required to formally establish SLCO4A1 as the PCS entry route, but the convergence of substrate class overlap, immune cell expression, and transcriptional suppression data provides a coherent mechanistic framework. LAT1/SLC7A5, the highest-expressed transporter gene in our dataset (**Figure 3**) is upregulated by PCS exposure but reflects ATF4-driven compensatory induction secondary to mTOR suppression rather than a direct entry route for PCS, as discussed in Section 4.

For SCFAs and other monocarboxylate GDBMs including hippurate, the primary entry route is distinct: monocarboxylate transporters MCT1 (SLC16A1) and MCT4 (SLC16A3) mediate proton-coupled transport of short-chain organic acids across immune cell membranes (36, 37). MCT1 is upregulated upon T cell activation and mediates butyrate import, enabling its intracellular HDAC inhibitory activity (61, 62). In our RNA-seq data, SLC16A1 (37) is also modestly upregulated by PCS at all three doses tested, while SLC16A3 (61), the primary lactate exporter in activated T cells (62), is the most dramatically downregulated transporter in the entire dataset (**Figure 3**). SLC16A3 suppression impairs lactate efflux, contributing to intracellular metabolic acidosis and the TCA cycle intermediate accumulation we observe in PCS-exposed cells. SLC7A5 expression in monocytes and macrophages also contributes to pro-inflammatory cytokine production downstream of leucine sensing (63) extending the relevance of this transport axis beyond the T cell compartment.

The feed-forward intracellular accumulation of PCS is not determined solely by enhanced influx. Equally important is the coordinate transcriptional suppression of efflux transporters that would otherwise clear aromatic conjugates from the cell. Three efflux transporters are significantly downregulated by PCS in our RNA-seq data: ABCC2 (MRP2), which preferentially exports sulfate and glucuronide conjugates (64),the exact chemical class to which PCS belongs, is downregulated in our RNAseq experiments; ABCC5 (MRP5), a broad organic anion efflux pump (65), is suppressed at all three concentrations including the lowest dose tested (10 μM) and SLCO4A1 (OATP4A1) (16),is downregulated at 50 and 100 μM (**Figure 3**). The mechanistic consequence is direct: PCS enters via SLCO4A1/OATP4A1, whose transcriptional suppression by intracellular PCS then impairs re-export of the same sulfated substrate class, a feed-forward retention loop. It is important to note that SLCO4A1 suppression and efflux transporter downregulation do not operate simultaneously but represent sequential phases of a retention program. Initial SLCO4A1-mediated entry precedes transcriptional suppression, and the subsequent coordinate downregulation of efflux transporters ABCC2, ABCC5, and SLC16A3 shifts the influx/efflux balance toward net intracellular retention rather than unlimited accumulation. The biologically relevant outcome is a sustained elevated intracellular steady-state concentration, sufficient to maintain AhR activation and its downstream consequences, rather than progressive unbounded accumulation. Simultaneously, ABCC2 and ABCC5, the efflux transporters most competent to handle sulfate-conjugated organic anions, are also transcriptionally suppressed. This creates a self-reinforcing intracellular trapping architecture that directly enables the AhR-driven pro-senescence program described in Section 4. Together, the downregulation of SLCO4A1 following initial entry, combined with suppression of efflux (ABCC2, ABCC5, MCT4) and upregulation of SLC16A1, establishes the intracellular microbiome metabolome not as the passive consequence of extracellular exposure but as the active product of a transcriptionally regulated transport program (**Figure 3**). Our findings are consistent with the Remote Sensing and Signaling Theory (RSST), which posits that multi-specific OAT, OATP, and MRP transporters function as a coordinated network mediating remote communication between organs, the gut microbiome, and circulating metabolites (16, 66–69). The RSST framework explicitly incorporates AhR activation by gut-derived uremic metabolites including PCS and indoxyl sulfate as part of a bidirectional signaling axis involving prostaglandins, cAMP, and other signaling molecules regulated through feedback mechanisms among OAT, OATP, and ABCC family transporters. We propose that PCS-driven dysregulation of SLCO4A1 and efflux transporters in CD4^+^ T cells represents a special case of RSST operating within the immune system, in which gut-derived uremic metabolites hijack the transporter network to enforce intracellular retention, chronic AhR activation, and immune dysfunction. This framework positions the intracellular GDBM compartment as a node within the broader RSST network and identifies transporter-mediated retention as a therapeutically actionable axis at the intersection of gut microbiome biology and HIV immunology.

## 4 Intracellular PCS activates AhR as the central pro-senescence axis

### 4.1 GCN2 as an intracellular amino acid sensor in immune cells: field context

GCN2 (EIF2AK4) is the only eIF2α kinase activated directly by amino acid insufficiency, sensing uncharged tRNAs through its histidyl-tRNA synthetase-like domain when cognate amino acids are depleted and aminoacyl-tRNA synthetases fail to charge their substrates (70, 71). Its role in immune regulation was established by Munn, Mellor, and colleagues, who demonstrated that IDO1-mediated tryptophan catabolism in antigen-presenting cells activates GCN2 in co-cultured T cells, suppressing T cell proliferation and promoting regulatory T cell differentiation through the downstream eIF2α/ATF4 axis (17, 18). This IDO/GCN2/Treg pathway defined the principle that amino acid insufficiency, whether by enzymatic catabolism or intracellular metabolic perturbation, can activate GCN2 in T cells with broad immunosuppressive and tolerogenic consequences. Complementary evidence from the transporter biology field showed that genetic disruption or pharmacological inhibition of LAT1 activates GCN2 and induces the downstream ATF4 target genes CHOP/DDIT3 and ASNS (72) establishing that leucine sensing via LAT1/mTORC1 and GCN2 activation are coupled nodes in T cell metabolic homeostasis. GCN2 activation by amino acid sensing also connects to the broader framework of dietary restriction-mediated longevity signaling: Gallinetti et al. demonstrated that GCN2 and TOR function as complementary amino acid sensors that cooperate to regulate longevity programs in response to nutrient availability (73) providing important context for how PCS-driven GCN2 activation may contribute to the accelerated aging phenotype of CD4^+^ T cells in dysbiotic disease states. Most directly relevant to our model, Hayashi et al. showed that JPH203, the selective LAT1 inhibitor, triggers GCN2-ATF4-CHOP signaling specifically in primary human T cells, suppressing cytokine production through an ISR-dependent mechanism (74) providing pharmacological validation that LAT1 inhibition activates GCN2/ISR in the exact cell type we study, and establishing the relevant downstream signaling cascade. In the context of our model, intracellular PCS, accumulated via SLCO4A1-mediated entry and retained through coordinate efflux suppression, is proposed to activate GCN2/ISR as a downstream consequence of metabolic disruption rather than through direct competitive displacement of leucine at the LAT1 transporter. The upstream trigger is intracellular GDBM burden and its perturbation of amino acid homeostasis and mTORC1 signaling; LAT1 upregulation observed in PCS-exposed cells represents the cell’s compensatory attempt to restore leucine supply and mTORC1 activity, not evidence of PCS entry via this route. Although the transcriptomic signature of ATF4, ATF3, and DDIT3/CHOP induction is consistent with ISR activation, it does not by itself identify GCN2 as the specific upstream eIF2α kinase, as PERK, PKR, or HRI could also contribute under conditions of metabolic or mitochondrial stress. GCN2 is favored as the primary candidate based on the established role of amino acid insufficiency signaling in T cells and the metabolic context of mTOR suppression and compensatory LAT1 induction observed in PCS-exposed cells, but direct measurement of p-GCN2 or GCN2-specific perturbation experiments will be required to confirm kinase identity.

### 4.2 The ISR futile loop: a self-amplifying mechanism

Consistent with this mechanistic framework, our RNA-seq data from PCS-exposed CD4^+^ T cells show transcriptomic signatures suggesting active GCN2/ISR engagement: ATF4 target genes, including ATF3, ATF4, and DDIT3/CHOP, are upregulated in a PCS dose-dependent manner, mirroring the ISR transcriptional program established in amino acid deprivation models (75, 76) (**Figure 3**). A key feature of ISR activation is the induction of SLC7A5/LAT1 by ATF4 as a homeostatic attempt to rescue leucine import and restore mTORC1 activity, a compensatory response to mTOR suppression rather than a mechanism of PCS entry (72, 75). This same compensatory LAT1 upregulation has been independently documented in aging CD4^+^ T cells by Jin et al., who showed that SLC7A5 is selectively elevated in activated T cells from older adults and sustains dysregulated mTORC1 activity through a cytoplasmic leucine-sensing mechanism (77) a finding that parallels the ATF4-driven LAT1 upregulation in our PCS model and suggests that dysregulated mTOR/GCN2 signaling with compensatory LAT1 induction may be a shared node across microbiome-driven and age-driven T cell dysfunction (78). In the PCS context, ATF4-driven SLC7A5 induction reflects a compensatory attempt to restore mTORC1 leucine sensing that is ultimately insufficient to reverse the metabolic arrest caused by intracellular GDBM burden. The persisting GCN2 activation, sustained eIF2α phosphorylation, maintained ATF4 translation, and further SLC7A5 upregulation constitute a cycle that reinforces the senescence-associated metabolic state as long as the intracellular PCS burden, maintained by SLCO4A1 entry and efflux suppression, remains unresolved. The ISR axis additionally converges on mTOR suppression through two independent arms: upstream nutrient deprivation through SLC1A5/ASCT2 downregulation reducing glutamine import (79) and direct mTORC1 inhibitory complex induction through NPRL2 upregulation at the protein level (80) providing convergent transcriptomic and proteomic evidence that mTOR suppression in PCS-exposed T cells (12) is mechanistically overdetermined.

### 4.3 AhR: the central pro-senescence axis

In parallel with the GCN2/ISR axis, our data suggest that intracellular PCS engages AhR as a direct agonist (12). AhR is a cytoplasmic ligand-activated transcription factor that, upon ligand binding, translocates to the nucleus and heterodimerizes with ARNT to drive transcription at xenobiotic response elements (XREs) (13, 81). PCS induced the full spectrum of AhR transcriptional targets including CYP1A1 and CYP1B1 alongside canonical AhR suppressors, with induction magnitude correlating with PCS dose and persisting across proliferative divisions (12). It is important to clarify the temporal relationship between AhR activation, proliferation, and senescence-associated arrest in this model. AhR target gene induction and cell proliferation are not mutually exclusive: in our experimental system, T cells are first activated and begin dividing before intracellular PCS accumulation reaches levels sufficient to drive the full downstream program. AhR activation during early proliferative divisions initiates a transcriptional reprogramming program that persists across cell cycles, consistent with epigenetic propagation of the AhR-driven transcriptional state. The downstream metabolic consequences, mTOR suppression, glycolytic collapse, and mitochondrial dysfunction, accumulate progressively over successive divisions as the five effector programs converge, ultimately triggering ISR activation and p16/p21-dependent cell cycle arrest as a late consequence rather than an immediate effect of AhR engagement. The resulting senescence phenotype therefore represents the endpoint of a temporally ordered program initiated by intracellular PCS accumulation, with AhR activation as the proximal trigger and ISR-driven cell cycle arrest as the terminal output. The functional consequences of AhR activation in this context are qualitatively distinct from those of the GCN2/ISR axis yet converge on the same immune fate. AhR activation in CD4^+^ T cells promotes FOXP3 expression and Treg polarization in a ligand-dependent manner (13, 82) consistent with the dose-dependent FOXP3 induction and Treg enrichment we observe both *in vitro* and in high-PCS donors ex vivo (12). AhR additionally activates TGF-β/SMAD signaling and Wnt/β-catenin programs, suppresses mTOR and glycolytic gene expression, and induces stemness-associated transcription factors TCF7 and LEF1 (12, 81, 83). In the context of immune aging, chronic AhR engagement by endogenous or microbial ligands has been proposed to promote immunosenescence through thymic involution, IDO-mediated immunosuppression, and reinforcement of the senescence-associated secretory phenotype (84–86). Recent work has further shown that AhR activation by uremic metabolites including IS promotes vascular inflammation in ESRD patients (87, 88) consistent with the p16/CDKN2A and p21/CDKN1A upregulation we observe alongside AhR target gene induction in PCS-exposed cells (12).The most recent Quintana laboratory review further positions AhR as a rehabilitated therapeutic target with the first AhR-activating drug recently approved for clinical use (89) underscoring the translational relevance of this pathway.

### 4.4 Dual pathway convergence and therapeutic implications

The AhR axis drives five downstream effector programs, TGF-β/SMAD, Wnt/β-catenin, Treg induction, Notch, and PTGS2/PGE_2_/CREM, that converge on mTOR suppression, glycolytic collapse, and mitochondrial dysfunction. This metabolic collapse in turn activates ISR as a downstream consequence, through global amino acid insufficiency and energy failure, driving translational reprogramming via eIF2α phosphorylation, selective ATF4-dependent induction of stress genes including DDIT3/CHOP, and cell cycle arrest via p16/CDKN2A and p21/CDKN1A induction. ISR is not a parallel independent axis but an obligate downstream effector of AhR-driven metabolic collapse: the ISR amplifies and sustains the senescence program initiated by AhR, but its activation requires the upstream metabolic disruption. This architecture has direct therapeutic implications: interventions targeting only ISR would address the downstream amplifier but leave the AhR-driven initiating programs intact, whereas interrupting intracellular PCS accumulation upstream, at the level of SLCO4A1-mediated entry or efflux transporter suppression, would disable AhR activation and all downstream consequences including ISR simultaneously. It is important to note that SLC16A3/MCT4 downregulation, one of the most pronounced transporter changes in our dataset, may itself contribute to mTOR suppression and stress pathway engagement through impaired lactate efflux and secondary intracellular acidification, independent of direct AhR transcriptional activity. Intracellular pH changes are recognized modulators of mTORC1 activity and can induce senescence-like metabolic states. We cannot formally exclude this as a contributing mechanism with our current data. However, MCT4 suppression is itself an established downstream consequence of AhR activation in other cellular contexts, suggesting that lactate accumulation and acidification may amplify rather than replace AhR-driven metabolic collapse, operating as a feed-forward component within the same hierarchical program. The full breadth of the transcriptional signature we observe, including canonical AhR targets CYP1A1 and CYP1B1, Treg induction, and Wnt/β-catenin reprogramming, exceeds what intracellular acidification alone would be predicted to produce and supports AhR as the primary upstream driver. Definitive dissection of these contributions will require AhR knockout experiments combined with intracellular pH measurement and lactate quantification in PCS-exposed CD4^+^ T cells. The proteomic data depicted in Da Silva et al. (12) provide convergent support: downregulation of FCHO1, GZMA, MAPK13, and SLC39A8 reflects the dismantling of TCR signaling, cytotoxic effector function, and antioxidant defense, while upregulation of NPRL2, TGFBRAP1, TLE3, BTG1, and ENSA documents the concurrent shift toward mTOR suppression, TGF-β signaling, epigenetic silencing, and cell cycle arrest, a coordinated proteomic signature of immunometabolic collapse consistent with AhR-driven activation of the full pro-senescence program in CD4 intracellular PCS accumulation in CD4^+^ T cells.

### 4.5 PTGS2/PGE_2_/EP2-EP4/CREM signaling: a convergent effector mechanism downstream of AhR activation

A further layer of senescence-associated signaling emerges downstream of AhR activation and warrants dedicated consideration. PCS-induced AhR directly transactivates PTGS2, encoding cyclooxygenase-2 (COX-2), the rate-limiting enzyme in prostaglandin E_2_ (PGE_2_) biosynthesis, via xenobiotic response elements in the PTGS2 promoter (13, 90), while simultaneously suppressing HPGD, the primary intracellular enzyme responsible for PGE_2_ degradation (91) (**Figure 3**). The coordinate induction of PGE_2_ production and suppression of its catabolism creates a feed-forward accumulation of PGE_2_ that amplifies the pro-senescence program established by AhR activation and its downstream metabolic consequences. PGE_2_ that exits the cell acts on EP2 and EP4 G-protein-coupled receptors at the cell surface, signaling that is well-established in the context of T cell dysfunction and immune suppression (92) EP2/EP4 engagement elevates intracellular cyclic AMP (cAMP) through Gs-coupled adenylyl cyclase activation, which in turn activates protein kinase A (PKA) (93). A key downstream transcriptional target of this cAMP/PKA cascade in T cells is CREM, the cAMP-responsive element modulator, which is significantly upregulated in our RNA-seq data at both 50 and 100 μM PCS (**Figure 3**). CREM is a repressor of IL-2 transcription and a well-characterized driver of T cell anergy and hyporesponsiveness in chronic disease contexts including systemic lupus erythematosus and HIV-1 infection, where its induction is associated with impaired effector T cell function and Treg enrichment (94, 95). The upregulation of CREM by intracellular PCS, mediated through the AhR-driven PTGS2/PGE_2_/EP2-EP4/cAMP/PKA cascade, thus represents a downstream effector arm of AhR activation that extends its reach beyond direct transcriptional targets into paracrine and autocrine immune suppression. Importantly, the PTGS2/PGE_2_/CREM cascade does not operate in isolation, it is mechanistically downstream of AhR, and its sustained activity is enabled by the metabolic collapse that AhR drives, creating a permissive intracellular environment for prolonged PGE_2_ signaling. AhR drives XRE-dependent transcriptional activation of five downstream effector programs: TGF-β/SMAD, Wnt/β-catenin, Treg induction, Notch, and PTGS2/PGE_2_/CREM, promoting Foxp3/Treg polarization, stemness, and T cell exhaustion (12, 81, 83). The resulting mTOR suppression, glycolytic collapse, and mitochondrial dysfunction then activate GCN2/ISR as a downstream metabolic consequence, driving translational reprogramming via eIF2α phosphorylation and ATF4/DDIT3 induction (75, 76). Their convergence on a shared functional outcome, Treg enrichment, p16/p21 induction, metabolic arrest, and impaired effector capacity, reinforces the conclusion that intracellular PCS accumulation activates a hierarchically organized, multi-layered senescence program unlikely to be reversed by targeting any single downstream effector (12). This architecture further strengthens the therapeutic argument for intervening upstream, at the level of SLCO4A1-mediated entry or efflux transporter suppression, as the point at which AhR activation and all downstream cascades including GCN2/ISR could be simultaneously disabled (21, 22).

## 5 Beyond PCS: generalizing the intracellular microbiome metabolome framework

PCS has served as the mechanistic prototype in this review, but the intracellular microbiome metabolome concept is explicitly generalized across metabolite classes and immune cell types. The structural logic of generalizability rests on two principles established in the preceding sections. Importantly, the intracellular microbiome metabolome framework does not predict uniform functional consequences across all aromatic GDBMs. Rather, the unifying principle is shared carrier-mediated entry and intracellular accumulation, while downstream functional impact varies in a metabolite-specific manner determined by conjugation state, transporter affinity, and intracellular routing, as detailed below. First, aromatic GDBMs derived from tyrosine (PCS, PCG), phenylalanine (PAG), and tryptophan (IS, IAA, IAA-OG) share the chemical character of amphipathic, conjugated organic anions, the substrate class recognized by OATP-family transporters including SLCO4A1/OATP4A1, predicting that these metabolites as a class will be subject to OATP-mediated cellular uptake, with efficiencies proportional to their transporter affinity and conjugation state (16). Second, sulfate and glucuronide conjugation, shared by PCS, IS, and PCG, renders these metabolites poor substrates for MRP2/ABCC2 and MRP5/ABCC5 efflux (64, 65) predicting that conjugated forms will be retained intracellularly more efficiently than non-conjugated precursors. Our metabolomic data from CD4^+^ T cells of PLWH are consistent with this prediction: PCS, PAG, PCG, IAA, IAAOG, and 3HPAGS all accumulate intracellularly and associate with overlapping metabolic pathway disruption signatures, despite their structural diversity as depicted in **Figure 1A**.

Among the remaining aromatic GDBMs detected intracellularly in our CD4^+^ T cell datasets, PAG and PCG represent intermediate cases that illuminate the boundaries of the framework. Our data show that intracellular PAG accumulates in CD4^+^ T cells of PLWH and correlates with metabolic pathway disruption signatures overlapping substantially with those of PCS (12) (**Figure 1**) consistent with shared GCN2/ISR engagement but potentially weaker given phenylalanine’s lesser role in mTORC1 activation relative to leucine. PCG shows an opposing metabolic correlation pattern, negatively correlating with pathways that PCS positively correlates (12) (**Figure 1**) suggesting that glucuronide versus sulfate conjugation may alter intracellular routing or AhR affinity in ways that require direct investigation. IAA accumulates intracellularly but shows minimal metabolic pathway correlation in our dataset (12) (**Figure 1**) consistent with lower OATP transporter affinity for the smaller, less conjugated indole acetic acid pharmacophore. This gradient, PCS and PAG with strong metabolic signatures, IAA with minimal, is consistent with OATP-substrate class predictions based on conjugation state and molecular amphipathicity.

Short-chain fatty acids represent a mechanistically distinct but conceptually parallel case that strengthens the generalizability argument from a different angle. Butyrate, propionate, and acetate enter immune cells via MCT1 (SLC16A1) rather than OATP-family transporters, and their intracellular effects are primarily mediated through HDAC inhibition rather than GCN2/ISR or AhR engagement (4, 5). The SCFA literature thus provides the established precedent that GDBMs can cross immune cell membranes via carrier-mediated transport and directly reprogram immune gene expression through intracellular enzymatic mechanisms, the foundational proof of concept for the intracellular microbiome metabolome framework. Whether SCFA and aromatic GDBM intracellular signals are additive, synergistic, or antagonistic in primary immune cells is an open question with direct relevance to understanding how the overall composition of the intracellular microbiome metabolome determines immune cell fate.

## 6 The intracellular microbiome metabolome and immune aging in chronic disease

Immunosenescence, the progressive deterioration of immune function with age, is characterized in the CD4^+^ T cell compartment by permanent cell cycle arrest, loss of proliferative capacity, acquisition of a senescence-associated secretory phenotype (SASP), and upregulation of the canonical cell cycle inhibitors p16/CDKN2A and p21/CDKN1A (12, 96, 97). These hallmarks are not confined to chronological aging: they are recapitulated prematurely in the context of chronic inflammatory diseases, including HIV-1 infection, CKD, and inflammatory bowel disease, where persistent immune activation, gut dysbiosis, and elevated circulating GDBMs converge (11). In PLWH receiving effective ART, CD4^+^ T cells exhibit accelerated senescence phenotypes, elevated p16 expression, reduced proliferative capacity, mitochondrial dysfunction, and transcriptional programs resembling those of naturally aged T cells in HIV-negative elderly individuals, despite durable viral suppression (98–102). The upstream drivers of this accelerated immune aging beyond viral factors remain incompletely defined, limiting the development of targeted interventions. The intracellular microbiome metabolome framework provides a mechanistically grounded candidate: gut dysbiosis-driven GDBM accumulation within CD4^+^ T cells activates AhR, engaging five downstream effector programs that collectively drive mTOR suppression, metabolic collapse, and GCN2/ISR activation, directly inducing p16 and p21 and offering a cell-autonomous explanation for immune aging that operates independently of active viral replication.

The clinical relevance of this framework is most directly illustrated in immunological non-responders (INRs), virally suppressed PLWH who fail to restore normal CD4^+^ T cell counts despite effective ART and represent a population in which the features of premature immune senescence are most pronounced (98, 99). Our previous work demonstrated that PCS is selectively enriched within CD4^+^ T cells of INRs compared with immunological responders across three independent cohorts recruited in Russia, Cleveland, and San Francisco (11). More recent data, from an independent Atlanta cohort of virally suppressed PLWH immune responders, show that cell-associated PCS concentrations stratify CD4^+^ T-cell phenotype, metabolic pathway activity, and transcriptional programs in a dose-dependent manner across four PCS quartile groups (no PCS, low, medium, and high), with high cell-associated PCS associated with p16/p21 induction, Treg enrichment, TEMRA contraction, AhR pathway activation, and TCA cycle intermediate accumulation (12). The convergence of *in vitro* mechanistic data with ex vivo clinical observations across independent cohorts strengthens the inference that intracellular PCS accumulation is not merely a correlate of immune dysfunction but a plausible contributor to its progression.

Although the clinical evidence presented here derives primarily from HIV-1 infection, the intracellular microbiome metabolome framework is broadly applicable to other conditions characterized by gut dysbiosis and elevated aromatic GDBMs. In CKD, plasma PCS and IS reach concentrations of 100–600 μM, substantially higher than in PLWH, and are associated with impaired T cell proliferation, reduced vaccine responses, and accelerated cardiovascular immune aging (10, 15, 19). The immune dysfunction in CKD mirrors the phenotype we observe *in vitro* and ex vivo in high-PCS in PLWH: reduced T cell effector capacity, Treg enrichment, AhR pathway activation, and mitochondrial stress. In normal aging, gut dysbiosis with depletion of SCFA-producing commensals and enrichment of proteolytic taxa is well documented and increases progressively after the sixth decade (10). Whether the rising GDBM burden of normal aging contributes to immunosenescence through intracellular accumulation mechanisms paralleling those described here for disease states is an open question that longitudinal metabolomic profiling of immune cells across the human lifespan could address.

Across the clinical contexts described INRs during treated HIV infection, CKD, and aging, mitochondrial dysfunction is a consistently reported feature of CD4^+^ T cell senescence (11, 98, 100, 103–105). Our proteomic data identify a coordinated mitochondrial stress response in PCS-exposed T cells: upregulation of the mitochondrial translation elongation factor GFM1, the heme biosynthesis enzyme FECH, and the mitochondrial quality control protease HTRA2, alongside downregulation of MCT4 (SLC16A3) impairing lactate efflux and TCA cycle intermediate accumulation ex vivo (12). This mitochondrial stress signature is consistent with PCS-driven bioenergetic failure operating downstream of AhR activation and upstream of GCN2/ISR engagement. The convergence of transporter disruption, AhR signaling, mitochondrial stress, and GCN2/ISR activation on a shared metabolic collapse phenotype suggests that intracellular GDBM accumulation does not activate isolated pathways but rather destabilizes the interconnected metabolic and signaling network that maintains T cell homeostasis.

## 7 Therapeutic implications

The framework developed in this review rests on a foundation of transcriptomic, proteomic, and metabolomic associations rather than direct causal proof. The most important outstanding experimental priority is therefore establishing causality: does genetic or pharmacological disruption of SLCO4A1-mediated PCS entry in primary CD4^+^ T cells abolish intracellular PCS accumulation, prevent AhR activation and its downstream programs, and rescue the senescence transcriptional program including GCN2/ISR? Our data support this model, but the essentiality considerations — AhR for physiological T cell differentiation and GCN2 for the normal stress response — mean that pharmacological inhibition of any single downstream node will produce confounding effects independent of PCS. The cleanest genetic approach to establishing entry-dependent causality is CRISPR-Cas9 knockout of SLCO4A1 in post-activation CD4^+^ T cells, with intracellular PCS quantification by LC-MS/MS as the primary entry readout, AhR target gene induction as the pathway readout, and p16/CDKN2A and p21/CDKN1A induction as the primary downstream senescence readout, bypassing proliferation-based assays entirely. If SLCO4A1 knockout abolishes intracellular PCS accumulation and prevents AhR target gene induction, this would establish SLCO4A1-mediated entry as the obligate upstream event and exclude extracellular receptor-mediated AhR activation as the primary mechanism.

Throughout this review, we have advanced the hypothesis that AhR functions as the central and sufficient driver of the PCS-induced senescence program, with all five downstream effector arms, TGF-β/SMAD, Wnt/β-catenin, Foxp3/Treg induction, Notch dysregulation, and PTGS2/PGE_2_/CREM, converging on mTOR suppression, glycolytic collapse, and GCN2/ISR activation as direct consequences of AhR-dependent transcriptional reprogramming. A critical and directly testable corollary of this hypothesis is that AhR knockout in PCS-exposed primary CD4^+^ T cells should fully rescue the senescence program. CRISPR-Cas9 knockout of AhR in post-activation CD4^+^ T cells exposed to PCS would provide a definitive test of this prediction: if AhR is both necessary and sufficient for the downstream program, its genetic ablation should abolish p16/CDKN2A and p21/CDKN1A induction, restore mTOR activity and glycolytic gene expression, prevent GCN2/ISR engagement, and collapse Treg enrichment, despite continued intracellular PCS accumulation through the intact SLCO4A1 entry mechanism. This experimental design therefore directly tests whether the five effector programs and their metabolic consequences are AhR-dependent or whether PCS additionally engages AhR-independent senescence pathways, such as direct CoA pathway perturbation through phenylacetyl-CoA intermediate accumulation, or GCN2 activation by metabolic stress operating upstream of or in parallel with AhR-dependent transcriptional collapse. A partial rescue in AhR-null PCS-exposed cells, defined as persistent GCN2/ISR engagement or residual p16/p21 induction in the absence of AhR target gene induction, would identify AhR-independent contributions to the senescence phenotype and require revision of the unified AhR-centric model presented here. Conversely, complete rescue in AhR-null cells would validate the central claim of this review: that AhR is the obligate and sufficient intracellular mediator of PCS-driven CD4^+^ T cell immune aging, and that its upstream activation by intracellular PCS, rather than any parallel stress pathway, is the mechanistic bottleneck at which therapeutic intervention would be most consequential. Together, the SLCO4A1 and AhR knockout experiments constitute a two-step causal hierarchy: the first establishes that intracellular accumulation through SLCO4A1 is required for the phenotype, the second establishes whether AhR is necessary and sufficient for translating that accumulation into the full senescence program. This experimental program, which is achievable with current primary CD4^+^ T cell gene editing technology (106, 107) represents the critical next step for the field.

A second question of direct therapeutic relevance is whether the damage caused by intracellular GDBM accumulation can be reversed once it is established. The concern is that PCS entry through SLCO4A1, combined with suppression of efflux transporters, creates a self-reinforcing loop that may progressively lock the cell into a dysregulated transporter state. More troubling is the epigenetic dimension: our proteomic data show upregulation of TLE3, PHC1, and BTG1, proteins involved in chromatin remodeling and transcriptional silencing, suggesting that sustained intracellular GDBM exposure induces chromatin-level changes that may maintain the senescence program even after the metabolite burden is reduced. In other words, removing the trigger may not be sufficient to reverse the damage if the epigenetic state has already been reset. Whether removing the PCS signal, by reducing circulating PCS through microbiome-directed interventions or by blocking cellular entry at SLCO4A1, can reverse established p16/p21 induction, restore transporter expression to baseline, and rescue mitochondrial function is not known. These experiments would also clarify whether the epigenetic layer constitutes a point of no return that requires senolytic rather than preventive strategies (42, 96, 108).

Beyond these immediate experimental priorities, the intracellular microbiome metabolome framework raises a set of broader questions organized in the next section by research layer, mechanistic, translational, and therapeutic, to provide a structured roadmap for the community.

## 8 Outstanding questions in intracellular microbiome metabolome biology

The mechanistic specificity of the intracellular microbiome metabolome framework identifies three distinct therapeutic intervention layers that the receptor-centric model did not make accessible. The first and most upstream layer targets microbial metabolite production: reducing the output of PCS, IS, and PAG from the gut microbiome through dietary substrate restriction, modulation of proteolytic microbial populations, or inhibition of the microbial enzymes responsible for p-cresol and indole generation tyrosine lyase and tryptophanase respectively. These strategies address the problem at its biological source and would reduce both plasma and intracellular GDBM burden, but their immune-specific efficacy requires validation.

The second layer targets cellular entry and retention: the SLCO4A1/OATP4A1 entry transporter and the efflux transporter network. SLCO4A1 blockade, using selective OATP inhibitors derivatives that are established pharmacological tools in the transporter field, could prevent initial PCS entry without disrupting the essential LAT1/leucine axis required for T cell proliferation, offering a mechanistic advantage over LAT1-directed approaches. Induction of efflux transporter expression, particularly ABCC2 and ABCC5, could interrupt the intracellular retention loop independently of entry blockade. The efflux transporter approach is conceptually attractive because it would clear existing intracellular GDBM burden rather than merely preventing new accumulation.

The third layer targets intracellular signaling downstream of accumulation: the AhR axis and its downstream GCN2/ISR consequences. ISRIB, a small molecule that restores cap-dependent translation downstream of eIF2α phosphorylation by stabilizing the eIF2B guanine nucleotide exchange factor (109), could in principle interrupt the downstream ISR component without affecting upstream intracellular PCS accumulation or AhR activation. AhR antagonists including StemRegenin-1 and related compounds modulate AhR-driven Treg induction and have been explored in ex vivo HSC expansion and immune reconstitution contexts (89). Importantly, neither ISRIB nor AhR antagonists alone would be expected to fully rescue the PCS-driven immunometabolic phenotype, because they address downstream effectors rather than the upstream intracellular PCS accumulation that drives AhR activation, reinforcing the therapeutic logic that SLCO4A1-level intervention is the most mechanistically complete strategy. The intracellular microbiome metabolome framework thus converts a descriptive clinical observation, that gut dysbiosis associates with immune aging, into a specific, layered, and pharmacologically testable mechanistic program.

## Data Availability

Bulk RNA-seq data are deposited in GEO under accession GSE329693. Proteomics data are deposited in ProteomeXchange/PRIDE under accession PXD077610. Metabolomics data are deposited in the Metabolomics Workbench (Study ID ST004747; DOI: dx.doi.org/10.21228/M86V8D).
